# Supplementary material for: Clinical Outcomes in Older Patients Aged over 75 Years Who Underwent Early Surgical Treatment for Pyogenic Vertebral Osteomyelitis
Source: J Clin Med. 2021 Nov 22;10(22):5451. doi: 10.3390/jcm10225451 (PMC8619355; doi:10.3390/jcm10225451)
Supplement: Supplementary file 1 [file jcm-10-05451-s001.zip › jcm-1410735-supplementary.pdf]

Supplementary Materials

Supplementary Table S1. Predictors of recurrence and mortality in the surgical cohort: univariable analysis.

Variables	Category	Recurrence			One-Year Mortality		
		Odds Ratio	95% CI	*p*-Value	Odds Ratio	95% CI	*p*-Value
Age	Per 1 year	0.93	(0.82–1.05)	0.234	1.04	(0.96–1.14)	0.344
	80–84 vs 75–79	0.31	(0.13–7.32)	0.008	0.66	(0.34–1.28)	0.217
	≥85 years vs 75–79	0.74	(0.25–2.21)	0.585	1.45	(0.60–3.49)	0.404
Sex	Male vs female	1.08	(0.49–2.40)	0.848	0.61	(0.31–1.18)	0.140
BMI (kg/m^2)		0.97	(0.86–1.09)	0.586	1.00	(0.91–1.10)	0.983
Charlson Comorbidity Index (CCI) score	Per 1 point	1.36	(1.16–1.60)	<0.001	1.58	(1.36–1.85)	<0.001
	2 and 3 vs 0–2	3.02	(0.77–11.78)	0.112	2.34	(0.90–6.13)	0.083
	Over 4 vs 0–2	10.28	(2.90–36.41)	<0.001	11.08	(4.51–27.24)	<0.001
Comorbidities	Cerebrovascular disease	1.50	(0.62–3.60)	0.366	0.69	(0.31–1.53)	0.361
	Myocardial infarction	1.22	(0.53–2.81)	0.646	2.20	(1.16–4.15)	0.015
	Congestive heart failure	1.34	(0.47–3.78)	0.585	3.48	(1.62–7.47)	0.001
	Chronic obstructive pulmonary disease	2.58	(0.94–7.09)	0.066	3.06	(1.30–7.18)	0.010
	Liver cirrhosis	3.14	(1.19–8.27)	0.021	5.92	(2.53–13.84)	<0.001
	End-stage renal disease	1.38	(0.93–2.04)	0.114	1.49	(1.09–2.05)	0.012
	Diabetes, uncomplicated	0.64	(0.23–1.80)	0.402	0.98	(0.50–1.92)	0.958
	Diabetes, complicated	5.88	(2.26–15.29)	<0.001	2.06	(0.89–4.75)	0.090
American Spinal Injury Association Scale grade	Grade A,B and C vs grade E	4.64	(1.69–12.77)	0.003	8.10	(3.42–19.17)	<0.001
	Grade D vs grade E	0.97	(0.36–2.67)	0.959	1.10	(0.501–2.404)	0.816
Primary region	Thoracic vs cervical	1.72	(0.36–8.30)	0.498	0.69	(0.27–1.77)	0.435
	Lumbosacrum vs cervical	2.76	(0.60–12.67)	0.193	1.18	(0.48–2.91)	0.724
	Multiple vs cervical	7.75	(0.84–71.31)	0.071	0.63	(0.06–6.17)	0.687
Presence of abscess	Epidural abscess	1.20	(0.26–5.48)	0.812	2.67	(0.60–11.92)	0.199
	Posterior to epidural space	0.58	(0.25–1.31)	0.188	0.81	(0.41–1.59)	0.533
	Anterior to epidural space	1.68	(0.74–3.84)	0.219	0.80	(0.44–1.46)	0.463
Number of infected vertebral bodies	Over 3 vs within 2 vertebral bodies	0.99	(0.43–2.28)	0.979	1.21	(0.62–2.36)	0.575
Severity of infection by Pola et al	Type A vs C	-	-	0.999	0.99	-	-
	Type B vs C	0.94	(0.21–4.34)	0.940	0.43	(0.09–1.92)	0.265
Causative organism	MRSA vs other gram-positive bacteria	4.67	(0.97–22.57)	0.055	1.05	(0.34–3.27)	0.929
	MSSA vs other gram-positive bacteria	1.52	(0.28–8.38)	0.630	1.51	(0.51–4.48)	0.454
	Gram negative vs other gram-positive bacteria	1.54	(0.31–7.54)	0.597	1.06	(0.38–2.95)	0.918

	Others or unidentified vs other gram-positive bacteria	1.22	(0.16–9.33)	0.850	1.56	(0.45–5.43)	0.488
	MRSA vs no MRSA	3.32	(1.47–7.48)	0.004	1.16	(0.54–2.50)	0.710
White blood cell (×10^9/L)	Initial	1.00	(1.00–1.00)	0.605	1.00	(1.00–1.00)	0.107
Erythrocyte sedimentation rate (ESR, mm/h)	Initial	1.00	(0.98–1.03)	0.929	1.01	(0.99–1.03)	0.196
C-reactive protein (CRP, mg/L)	Initial	1.01	(0.99–1.02)	0.360	1.00	(0.99–1.01)	0.633

Supplementary Table S2. Comparison of baseline patient characteristics of the two surgical cohorts before and after propensity score matching.

		Whole Surgical Cohort			Matched Surgical Cohort		
		Non-Instrumented Group	Instrumented Group	*p*-Value	Non-Instrumented Group	Instrumented Group	*p*-Value
**Number of Patients**		130	115		80	80	
Age	Median (interquartile ranges)	80 (79–83)	81 (79–82)	0.838	80 (78–82)	81 (79–83)	0.059
	75 to 79 years	44 (34)	38 (33)	0.979	32 (40)	27 (34)	0.296
	80 to 84 years	69 (53)	61 (53)		42 (53)	41 (51)	
	≥ 85 years	17 (13)	16 (14)		6 (8)	12 (15)	
Sex ratio (F : M)		86 : 44	73 : 42	0.661	59 : 21	52 : 28	0.230
BMI (kg/m2)		22.4 (21.2–24.5)	23.9 (21.0–26.3)	0.170	22.6 (21.5–25.1)	23.2 (20.8–25.7)	0.941
Charlson Comorbidity Index score	Median (interquartile ranges)	3.0 (1.0–4.0)	2.0 (1.0–4.0)	0.041	2.0 (1.0–4.0)	2.0 (1.0–4.0)	0.970
	0 and 1	37 (28)	50 (43)	0.047	28 (35)	29 (36)	0.982
	2 and 3	49 (38)	36 (31)		26 (33)	25 (31)	
	over 4	44 (34)	29 (25)		26 (33)	26 (33)	
Comorbidities	Cerebrovascular disease	27 (21)	26 (23)	0.727	14 (18)	15 (19)	0.837
	Myocardial infarction	36 (28)	29 (25)	0.661	22 (28)	25 (31)	0.603
	Congestive heart failure	22 (17)	11 (10)	0.092	12 (15)	10 (13)	0.646
	Chronic obstructive pulmonary disease	15 (12)	10 (9)	0.463	5 (6)	7 (9)	0.548
	Liver cirrhosis	17 (13)	9 (8)	0.183	9 (11)	8 (10)	0.798
	End-stage renal disease	41 (32)	27 (23)	0.160	24 (30)	24 (30)	1.000
	Diabetes			0.612			0.940
	Complicated *	60 (46)	52 (45)		36 (45)	38 (48)	
	Uncomplicated	22 (17)	15 (13)		14 (18)	14 (18)	
Bone Mineral density (g/cm²)	Spine	0.854 (0.732–1.010)	0.892 (0.777–0.981)	0.317	862 (755–931)	867 (783–934)	0.854
	Femur	0.645 (0.575–0.743)	0.700 (0.609–0.757)	0.101	654 (575–776)	710 (614–787)	0.460
American Spinal Injury Association Scale grade	Grade A,B and C	18 (14)	25 (22)	0.118	13 (16)	18 (23)	0.023
	Grade D	71 (55)	49 (43)		52 (65)	35 (44)	
	Grade E	41 (32)	41(36)		15 (19)	27 (34)	
Duration of intravenous antibiotics (days)		44 (42–56)	52 (45–63)	<0.001	54 (42–59)	52 (45–61)	0.224
Hospital stay (days)		52 (47–59)	54 (47–63)	0.084	53 (48–63)	54 (47–63)	0.710
Follow-up period		801 (478–1262)	1036 (597–1457)		855 ± 583	1050 ± 723	

* Complicated diabetes was defined as diabetes associated with damage of end organs including cardiovascular system, kidneys, eyes, and nervous system, and it included peripheral neuropathy, nephropathy, retinopathy, and peripheral artery disease.

Supplementary Table S3. Comparison of infection profiles of the two surgical cohorts before and after propensity score matching.

		Whole Surgical Cohort			Matched Surgical Cohort		
		Non-Instrumented Group	Instrumented Group	*p*-Value	Non-Instrumented Group	Instrumented Group	*p*-Value
Primary region	Cervical spine	10 (8)	23 (20)	0.022	8 (10)	12 (15)	0.812
	Thoracic spine	52 (40)	48 (42)		33(41)	30 (38)	
	Lumbosacrum	64 (49)	42 (37)		37 (46)	36 (45)	
	Multiple	4 (3)	2 (2)		2 (3)	2 (3)	
Presence of abscess	Epidural abscess	115 (88)	111 (97)	0.019	73 (91)	76 (95)	0.349
	Posterior to epidural space	96 (74)	91 (79)	0.332	58 (73)	63 (79)	0.357
	Anterior to epidural space	81 (62)	66 (57)	0.433	47 (59)	49 (61)	0.747
Number of infected vertebral bodies	within 2 vertebral bodies	95 (73)	77 (67)	0.296	61 (76)	52 (65)	0.118
	over 3 vertebral bodies	35 (27)	38 (33)		19 (24)	28 (35)	
Severity of infection by Pola et al	Type A	2 (2)	0 (0)	0.015	0 (0)	0 (0)	0.349
	Type B	13 (10)	4 (3)		7 (9)	4 (5)	
	Type C	115 (88)	111 (97)		73 (91)	76 (95)	
Causative organism	Staphylococcus aureus	52 (40)	47 (41)	0.586	28 (35)	38 (48)	0.373
	Methicillin resistant	24 (18)	24 (21)		11 (14)	20 (25)	
	Methicillin sensitive	28 (22)	23 (20)		17 (21)	18 (23)	
	Other gram-positive bacteria	15 (12)	15 (13)		11 (14)	9 (11)	
	Gram-negative bacteria	46 (35)	45 (39)		32 (40)	28 (35)	
	Others or unidentified	17 (13)	8 (7)		9 (11)	5 (6)	
White blood cell ($\times 10^9$/L)	Initial	12679 (10105–15436)	13060 (10330–16799)	0.155	12223 (9962–15747)	13309 (10268–16799)	0.302
Erythrocyte sedimentation rate (ESR, mm/h)	Initial	62 (47–71)	69 (53–75)	0.008	68 (52–74)	68 (50–72)	0.797
C-reactive protein (CRP, mg/L)	Initial	69 (58–83)	75 (67–88)	0.036	70 (63–86)	77 (62–93)	0.726

Supplementary Table S4. Comparison of Surgical profiles and treatment outcome of the two surgical cohorts before and after propensity score matching.

		Whole Surgical Cohort			Matched Surgical Cohort		
		**Non-Instrumented Group**	**Instrumented Group**	***p*-Value**	**Non-Instrumented Group**	**Instrumented Group**	***p*-Value**
Time between diagnosis and surgery	<2 weeks	95 (73)	89 (77)	0.207	68 (85)	61 (76)	0.312
	2 to 4 weeks	20 (15)	20 (17)		10 (13)	14 (18)	
	4 to 6 weeks	15 (12)	6 (5)		2 (3)	5 (6)	
Surgical approach	Anterior	6 (5)	18 (16)		4 (5)	18 (23)	
	Posterior	124 (95)	89 (77)		76 (95)	54 (68)	
	Anterior + Posterior		8 (7)			8 (10)	
Surgery related complications	Overall reoperation	10 (8)	11 (10)	0.601	7 (9)	8 (10)	1.000
	Implant failure		8 (7)			4 (5)	
	Wound dehiscence	7 (5)	9 (8)		3 (4)	6 (8)	
Medical events after surgery	At least one of the following complications	39 (30)	25 (22)	0.142	16 (20)	14 (18)	0.685
	Cardiac event	11 (8)	7 (6)		3 (4)	4 (5)	
	Respiratory complication	25 (19)	15 (13)		11 (14)	8 (10)	
	Cerebrovascular complication	4 (3)	4 (3)		1 (1)	2 (3)	
	Pulmonary embolism	8 (6)	4 (3)		3 (4)	2 (3)	
Recurrence	Occurrence	19 (15)	11 (10)	0.229	8 (10)	10 (13)	0.617
	Interval between initial diagnosis and recurrence (days)	39 (23–57)	142 (96–194)		34 (25–52)	139 (94–206)	
Mortality	90-day mortality	14 (11)	10 (9)	0.586	6 (8)	8 (10)	0.576
	1-year mortality	36 (28)	20 (17)	0.055	18 (23)	18 (23)	1.000

Supplementary Table S5. Causative organisms of the three groups.

Types of Bacteria		Conservative Group	Non-Instrumented Group	Instrumented Group
Staphylococcus aureus	Methicillin-Resistant Staphylococcus aureus	40	24	24
	Methicillin-Sensitive Staphylococcus aureus	38	28	23
Other gram-positive bacteria	Coagulase-negative staphylococci	10	7	5
	Streptococcus pneumoniae	2	0	2
	Streptococcus agalactiae	3	4	4
	Streptococcus viridans	3	1	0
	Enterococcus faecalis	5	1	2
	Enterococcus faecium	0	2	2
Gram-negative bacteria	Escherichia coli	30	22	17
	Pseudomonas aeruginosa	17	8	11
	Acinetobacter baumannii	5	2	1
	Klebsiella pneumoniae	6	7	9
	Salmonella enterica	4	6	4
	Enterobacter cloacae	3	0	1
	Enterobacter aerogenes	2	0	0
	Other Enterobacteriaceae	3	1	2
Others	Anaerobe	5	1	0
	Mixed infection	5	4	5
Unidentified		13	12	3

The causative organisms were categorized as “unidentified” when initial culture results were suspected to be contaminated from subsequent antibiotic response or when they showed different culture results from subsequent cultures.
